# Traditional agricultural practices and the sex ratio today

**DOI:** 10.1371/journal.pone.0190510

**Published:** 2018-01-16

**Authors:** Alberto Alesina, Paola Giuliano, Nathan Nunn

**Affiliations:** 1 Department of Economics, Harvard University, Cambridge, MA, United States of America; 2 NBER, Cambridge, MA, United States of America; 3 Anderson School of Management, UCLA, Los Angeles, CA, United States of America; University of Turku, FINLAND

## Abstract

We study the historical origins of cross-country differences in the male-to-female sex ratio. Our analysis focuses on the use of the plough in traditional agriculture. In societies that did not use the plough, women tended to participate in agriculture as actively as men. By contrast, in societies that used the plough, men specialized in agricultural work, due to the physical strength needed to pull the plough or control the animal that pulls it. We hypothesize that this difference caused plough-using societies to value boys more than girls. Today, this belief is reflected in male-biased sex ratios, which arise due to sex-selective abortion or infanticide, or gender-differences in access to family resources, which results in higher mortality rates for girls. Testing this hypothesis, we show that descendants of societies that traditionally practiced plough agriculture today have higher average male-to-female sex ratios. We find that this effect systematically increases in magnitude and statistical significance as one looks at older cohorts. Estimates using instrumental variables confirm our findings from multivariate OLS analysis.

## Introduction

The sex ratio—the proportion of males relative to females in a population—exhibits remarkable heterogeneity across countries. In particular, in many countries around the world, there is a disproportionate number of males relative to females. This well-recognized fact led Amartya Sen to declare in 1990 that more than 100 million women were “missing” [1, 2]. Examining data on the global cross-country distribution of the average male-to-female sex ratio between 2000 and 2009, one finds that the sex ratio varies widely. For example, for children under age 5, it ranges from just under 1 for a number of countries (e.g., Rwanda, Angola, Togo, etc.) to 1.10 in China. S1 Fig shows the distribution of sex ratios for different age groups. As it is apparent from the figure, most countries in our sample have a sex ratio around 105. A non-trivial fraction of our sample (53 countries out of 153) has a sex ratio higher than 105. These countries belong to all continents (47% from Europe, 36% from Asia, 10% from Africa and 7% from North and South America). The sex ratio goes down with age but still shows a large amount of variation for all age groups. Although China is particularly well-known for having a male-biased sex-ratio, there are many other countries that also have sex ratios that are strongly male-biased, e.g., South Korea (1.08), India (1.08), Albania (1.07), Georgia (1.06), Singapore (1.06), Jordan (1.06), etc.

A range of factors underlying the noticeably larger proportion of males in many countries has been well studied. The correlates of the sex-ratio at birth include parental well-being [3], birth order [4], and fertility [5]. The correlates of the sex ratio after birth have also been widely studied and include economic opportunities for women [6, 7], patriarchy and kinship structures [8, 9], and disease burden [10]. For a review of the literature see [11, 12].

In this paper, we test the hypothesis that differences in the sex ratio are determined, in part, by differences in the agricultural technologies traditionally used by a society’s pre-industrial ancestors. Earlier research by Ester Boserup [13] has suggested that the use of plough agriculture generated a division of labor where men worked in the fields and women specialized in work within the home. This is because the use of the plough in agriculture requires significant upper body strength, grip strength, and bursts of power to pull the plough itself or control the animal that pulls it. This can be contrasted to shifting cultivation, where women tended to participate as actively in agriculture as men. This form of agriculture tended to use the hoe and digging stick, and, although it required very hard work, did not require the same level of physical strength as plough agriculture. In addition, shifting cultivation was compatible with child care, in contrast to plough agriculture, where the presence of large animals made child care dangerous.

According to Boserup, differences in agricultural practices led to the appearance of different norms about the role of women in society. Societies featuring plough agriculture exhibited weaker beliefs about the equality of men and women in society, which has persisted until today, affecting differences in female labor force participation [14]. An additional consequence of traditional plough agricultural is that girls came to be valued less than boys. In turn, this resulted in the emergence of the custom of the dowry, where girls’ parents are required to make a large payment to the newly formed family and/or the groom’s family. In societies without the plough, a bride price was more likely to arise, where a large payment is made from the groom’s family to the bride’s. These differences in marriage customs further reinforced societies preference for boys over girls in societies with plough agriculture. This male-preference is likely to result in male-biased sex ratios of children. Although this could occur through selective abortion or infanticide in more extreme cases, the most likely mechanism is through differential access to nutrition, health care, and similar resources, which result in differential mortality rates for boys and girls.

In Boserup’s own words: “In communities where girls live in seclusion, and a large dowry must be paid when they marry, parents naturally come to dread the burden of having daughters. In some of the farming communities in Northern India, where women do little work in agriculture and the parents know that a daughter will, in due course, cost them the payment of a dowry, it was customary in earlier times to limit the number of surviving daughters by infanticide.” (p. 37) Boserup continues to explain that “This practice has disappeared, in its outward forms, but nevertheless the ratio of female to male population in these districts continues to be abnormal compared to other regions of India. […] The only plausible hypothesis is that mortality among girls was higher than among boys. […] the persistence of socio-cultural factors are believed to be largely responsible for the excess of female mortality over the male. For example, one of these socio-cultural factors seems to be a widespread supposition that milk is not good for girls, but is good for boys. […] There is also a tendency to care more for sick boys than for sick girls.” (p. 37)

Recent research has shown that parents often treat boys and girls differently. For example, differential allocation of food to sons relative to daughters can explain the global distribution of stunting [15], and it is plausible that they also have effects on mortality rates, thus affecting the sex ratio. Evidence from India shows that boys are breastfed longer [16]. In the same country girls with congenital heart disease are less likely to have a surgery than boys [17]. More generally, parents may be more willing to spend money on healthcare on their son than their daughter. A son preference will also cause parents to have a sex-biased stopping rule [16]. For example, if a family that could only financially support four children had four daughters, a male gender bias would make this family more likely to have another child in an attempt to have at least one son. Thus, families that consist of more daughters tend to be larger and living beyond their means.

In this paper, we test whether a tradition of plough agriculture is associated with differences in the male-to-female sex ratio. Our focus on a specific historical determinant of the sex-ratio is not meant to imply that other factors are irrelevant in determining the sex ratio. The sex ratio can be affected by a host of factors at conception (primary sex ratio), during pregnancy (secondary sex ratio), and after birth (tertiary sex ratio) [18, 19]. A large literature in biology and medicine has studied the determinants of primary and secondary sex ratios [11]. Sociologists and economists have examined a wide range of explanations for differences in tertiary sex ratios, including income [20–24], intra-household allocation of resources across children [25, 6], different preferences about the gender of the children [7] and social norms ([26, 27, 28, 29] for the case of China). Our analysis accounts for those alternative determinants of the sex ratio through the use of an instrumental variables estimation strategy.

The hypothesis we test relies on the assumption that the historical adoption of the plough continues to affect the sex ratio today, decades after most societies have moved out of agriculture. Recent evidence in economics shows that historical events can have highly persistent effects on beliefs and values. For example, [30] show that areas that were more anti-semitic during the Black Death were also more anti-Semitic during the early 20^th^ century. It has been shown that the slave trade in Africa, which occurred roughly from 1500 until 1850, not only continues to affect economic outcomes like per capita income [31], but also a range of cultural characteristics, such as ethnic diversity [32], trust [33], polygamy [34], and gender roles [35]. Differences in geographical characteristics related to the suitability of crops with different caloric yields appear to be responsible for the evolution of more patient time preferences [36]. Evidence of such long-term persistence is also not confined to the economics literature. The classic work of [37] on the culture of honor traces the effects of ancestral herding on the culture of honor and violence that one observes in the U.S. South today, while, more recently, [38] document how historical specialization in wheat, rather than wet rice agriculture in China, led to a greater prevalence of individualism today.

## Material and methods

### Variable construction and definition

#### Ancestral plough use

Our analysis examines the cross-country relationship between ancestral plough-use and the male-to-female sex ratio in the period after World War II. The measure of ancestral plough agriculture that we use is constructed from the variable *v*39 of the *Ethnographic Atlas* [39], which is a dataset that contains information on the traditional characteristics of 1,265 ethnic groups. The validity of the data from the *Ethnographic Atlas* has recently been verified by [40] in a study that links *Ethnographic Atlas* data to the same measures from contemporary individual-level survey data. The study finds a strong correlation between the coding of cultural practices in the *Ethnographic Atlas* and the prevalence of the same practices today. Similarly, [14] also documented that the measure of female participation in agriculture from the *Ethnographic Atlas* correlates very strongly with female labor force participation today.

The variable *v*39 classifies each ethnic group as being in one of the following three categories: (1) the plough was absent, (2) the plough existed at the time the group was observed, but it was not aboriginal, and (3) the plough was aboriginal, having existed prior to contact. Using this information, we construct an indicator variable that equals one if the plough was ever adopted during the pre-industrial period (whether aboriginal or not) and zero otherwise. We collapse the three categories of traditional plough use into one indicator variable because of the small number of ethnicities (only 18) that fall into the second category.

To construct measures of historical plough use at the country level, we use the same procedure as in [14]. The procedure, which is described in more detail in [16], uses the geographic distribution of 7,612 languages and dialects across the globe today, obtained from the 16^th^ edition of the *Ethnologue*: *Languages of the World* (Lewis, 2009), together with the *Landscan 2000* database, which reports estimates of the world’s population in 2000 at the 30 arc-second (roughly 1 km) grid cell level globally. We combine the *Ethnologue* data, which are in the format of a shape file, with the *Landscan* data, which are in the format of a raster file, to obtain an estimate of the location and number of people speaking all languages and dialects today.

To illustrate the procedure, we follow [14] and use the country of Ethiopia as an example. S2 Fig shows a map of the land inhabited by different ethnic groups; that is, groups speaking different languages and dialects. Each polygon, which is from *Ethnologue*, represents the approximate border of a group. The map also shows the *Landscan* estimate of the population of each (raster) cell within the country. A darker shade indicates more people living in the cell.

By matching each of the 7,612 *Ethnologue* language groups to one of the 1,265 *Ethnographic Atlas* ethnic groups, we create an estimate, for each 1km grid-cell globally, of whether the population’s ancestors used the plough in traditional pre-industrial agriculture. S3 Fig shows this for Ethiopia. We combine the estimate of ancestral plough use among all individuals across the world with information on modern country borders to construct an estimate of the fraction of the population currently living in a country with ancestors that engaged in traditional plough agriculture. S4 Fig displays the resulting country-level ancestral plough-use variable visually for each country in the World.

#### Outcome variables

The outcome of interest is the male-to-female sex ratio, measured as the number of males per 100 females. We construct our measure of male-to-female sex ratio by taking the average of quinquennial data from the *Demographic Yearbook* of the United Nations from 1960–2010. The *Demographic Yearbook* contains information on the sex ratio of different age groups. We examine the sex ratio of different groups of children, including the sex ratio: at birth, for ages 0–1, for ages 0–4, and for ages 5–14.

#### Control variables

All regressions include the following control variables from the contemporary period: the level of economic development, measured by the natural log of a country’s real per capita GDP, and expressed non-linearly with a second-order polynomial; fertility; and infant mortality. All controls are constructed by taking the average from 1960–2010. We also include continent fixed effects, which control for broad differences in the sex ratio that vary across large geographical regions.

We also control for a rich set of ethnographic controls that measure pre-industrial characteristics of a country’s ancestors. These are constructed using data from the *Ethnographic Atlas* and the same procedure that was used to construct the ancestral plough measure. The controls include the presence of large domesticated animals; the presence of a tropical or subtropical climate; a measure of overall agricultural suitability; the number of levels of political hierarchy beyond the local community (political complexity); and a measure of the complexity of settlement patterns (economic complexity).

The presence of large domesticated animals is measured using variable *v40*. In the original classification, the nature of animal husbandry is classified into seven categories: absence of large domesticated animals; pigs are the only large animals; presence of sheep and/or goat without any larger domesticated animals; presence of equine animals; presence of deer; presence of camels, alpacas or llamas; and presence of bovine animals. We create an indicator variable that equals one if any type of large domesticated animals were present in the society. Economic development is measured using variable *v*30. This variable classifies groups into one of eight different types of settlements: (1) nomadic or fully migratory, (2) semi-nomadic, (3) semi-sedentary, (4) compact but not permanent settlements, (5) neighborhoods of disperse family homesteads, (6) separate hamlets forming a single community, (7) compact and relatively permanent settlements and (8) complex settlements. We construct a variable that takes on integer values, ranging from 1 to 8, that is increasing with settlement complexity. Political complexity is measured using variable *v*33, which classifies the number of jurisdictional hierarchies that exist beyond the local community. The variable takes on values from 1 to 5, with 1 indicating no levels of hierarchy beyond the local community and 5 indicating four levels.

Data on fertility (number of children born per woman) and infant mortality (number of infant deaths per 1,000 live births) are taken from the same source as the sex ratio data, which is the *Demographic Yearbook* of the United Nations. Real per capita GDP measures are taken from the Maddison project and are expressed in 1990 international dollars.

We construct our geographical characteristics variables using information coming from the FAO’s Global Agro-Ecological Zones (GAEZ) v3.0 database [41]. The database reports the suitability for the cultivation of different crops for grid-cells 5 arc-minutes by 5 arc-minutes (approximately 56 km by 56 km) for the world. Using this, we calculate the proportion of land within a 200-kilometer radius of an ancestor’s location that is defined as being either tropical or subtropical. Information on the location of ancestral groups is taken from the *Ethnographic Atlas*.

#### Instrumental variables

Our baseline analysis uses instrumental variables to estimate the causal effect of ancestral plough use. For instruments, we use information on the suitability of growing different crops, some of which benefitted from the introduction of the plough more than others. Pryor [42] classifies crops into those that benefitted more from the plough (called plough-positive crops) and those that benefitted less from the plough (called plough-negative crops). Plough-positive crops, which typically require land preparation over a large surface area and during a very short period of time, include barley, wheat, rye, teff, and wet rice. Plough-negative crops, which tend to yield more calories per acre, have longer growing seasons, and can be cultivated on more marginal, rocky, and/or slopped land, include maize, sorghum, millet, tree crops, and root crops.

To construct instruments for the IV regressions, we first restrict attention to Old World cereals crops: wheat, barley and rye (plough positive), and foxtail millet, pearl millet and sorghum (plough negative). These crops are comparable on a number of dimensions: they require similar preparations for consumption, produce similar yields, and thus can support similar population densities. From the FAO’s GAEZ database, we extract the raster files for the relevant crops and, using information from the *Ethnographic Atlas* on the historical locations of ethnic groups, construct a measure of the fraction of land within 200 kilometers of the centroid of ancestral ethnic groups that can grow each plough positive crop and each plough negative crop. We then create an average measure of three plough-positive suitability measures and normalize this by overall suitability for the cultivation of crops. This is the first instrument. The second instrument is the same measure but created as an average of the three plough-negative suitability measures also normalized by overall agricultural suitability.

### Descriptive analysis

**Fig 1:**
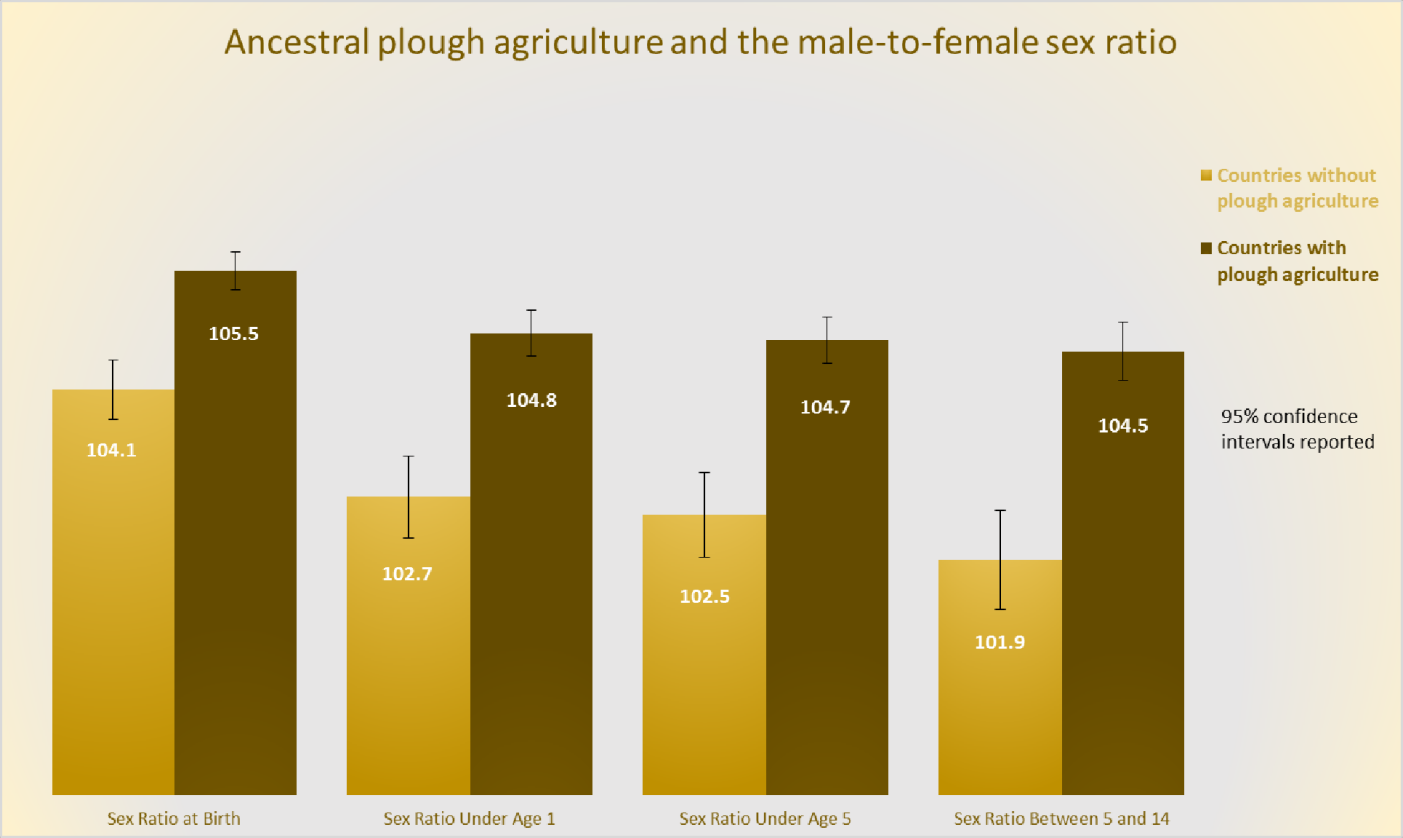
Countries with ancestors who used the plough show a higher male-to-female sex ratio today. The figure shows the average sex ratio (boys per 100 girls) in countries with ancestors that engaged in plough agriculture and in countries with ancestors that did not engage in plough agriculture for different age ranges. The sample includes 146 countries, 79 of which are countries that are categorized as traditionally engaging in plough agriculture and 67 as not traditionally engaging in plough agriculture. “Countries with plough agriculture” are countries for which 95% or more of its population has ancestors that belong to ethnic groups that traditionally engaged in plough agriculture. “Countries without plough agriculture” are countries for which 95% or more of its population has ancestors that belong to ethnic groups that traditionally did not engage in plough agriculture. The reported sex ratios are quinquennial averages from 1960–2000. The difference in the sex ratios between the two groups is significant at the 1% level for all age groups.

We begin our analysis by first examining the raw data. This is done visually in Fig 1, which reports the average sex ratio from 1960–2010 for two groups of countries. One is 79 countries for which more than 95% of the population has ancestors that used the plough traditionally and the other is 67 countries for which less than 5% of the population has ancestors that used the plough. From the figure, we see that for all age cohorts, the male-to-female sex ratio is greater among societies that traditionally engaged in plough agriculture. While this is true for the sex ratio at birth, it becomes stronger for older children. This is potentially explained by higher mortality rates among girls than boys.

The differences that we observe in the raw data are potentially explained by a host of other determinants other than traditional plough agriculture. Thus, we now turn to more formal multivariate OLS and IV estimates of the determinants of the sex ratio. In particular, the IV analysis addresses the possibility that societies with different gender norms historically may have been more likely to adopt technologies, like the plough, that reinforced the original biases. Before presenting the IV estimates, we first report, for comparisons, the multivariate OLS estimates of the relationship between ancestral plough agriculture and the sex ratio. These estimates account for a number of factors that may have their own effect on the sex ratio. The list of control variables included in the regression was described above.

The multivariate OLS estimates are reported in Panel A of Table 1. Each column of the table reports estimates for a different age cohort. The table reports coefficient estimates and robust standard errors, as well as Conley standard errors that adjust for non-independence of the observations [43, 44]. For our baseline estimates, we use the average geographical distance between a country’s ancestors to adjust for spatial dependence. As we discuss below, standard errors are similar to the use of a range of alternative methods to correct for non-independence using the Conley method.

**Table 1:** Sex ratio and ancestral plough use.

	(1)	(2)	(3)	(4)	(5)	(6)
	Sex ratio at birth	Sex ratio under age 1	Sex ratio age 0 to 4	Sex ratio age 5 to 14	PC, all sex ratios	PC, sex ratios 0–4 and 5–14
	Panel A: OLS estimates					
Mean (std. dev.) of sex ratio	104.8 (1.44)	103.7 (1.90)	103.5 (1.98)	103.2 (2.35)	0.00 (1.93)	0.00 (1.39)
**Ancestral plough use**	0.664*	1.040***	1.194***	1.744***	1.171***	0.948***
** **	**(0.337)**	**(0.386)**	**(0.414)**	**(0.509)**	**(0.414)**	**(0.296)**
	**[0.290]**	**[0.352]**	**[0.382]**	**[0.482]**	**[0.376]**	**[0.277]**
**Ancestral plough use (beta coefficient)**	0.204*	0.242***	0.267***	0.329***	0.273***	0.307***
	Panel B: Second stage of 2SLS estimates					
Mean (std. dev.) of sex ratio	104.8 (1.44)	103.7 (1.90)	103.5 (1.98)	103.2 (2.35)	0.00 (1.93)	0.00 (1.39)
**Ancestral plough use**	**0.741**	1.742***	1.912***	3.012***	1.832***	1.584***
** **	**(0.498)**	**(0.604)**	**(0.644)**	**(0.851)**	**(0.637)**	**(0.476)**
	**[0.477]**	**[0.585]**	**[0.647]**	**[0.867]**	**[0.634]**	**[0.481]**
**Ancestral plough use (beta coefficient)**	**0.232**	0.413***	0.435***	0.577***	0.427***	0.513***
	Panel C: First stage of 2SLS estimates. Dependent variable: Traditional plough use					
Plough-positive environment	0.741***	0.741***	0.741***	0.741***	0.741***	0.741***
	(0.088)	(0.088)	(0.088)	(0.088)	(0.088)	(0.088)
Plough-negative environment	0.258	0.258	0.258	0.258	0.258	0.258
	(0.172)	(0.172)	(0.172)	(0.172)	(0.172)	(0.172)
*F*-statistic (environment instruments)	42.03	42.03	42.03	42.03	42.03	42.03
Continent fixed effects	yes	yes	yes	yes	yes	yes
Mean (std. dev.) of ancestral plough use	0.63 (0.45)	0.63 (0.45)	0.63 (0.45)	0.63 (0.45)	0.63 (0.44)	0.63 (0.44)
Observations	152	152	152	152	152	152
R-squared	0.506	0.612	0.601	0.555	0.585	0.585

*Notes*: The unit of observation is a country. Coefficients are reported with robust standard errors in parenthesis. ‘‘Ancestral plough use” is the estimated proportion of citizens with ancestors that used the plough in pre-industrial agriculture. The variable ranges from 0 to 1. The dependent variables are the number of boys of a given age range per 100 girls, for the 1960–2009 period. The regressions include the historical and contemporary controls used in Table 1. The instruments comprise two variables: one measuring the ancestral suitability of the environment for plough-positive crops (the average fraction of ancestral land that was suitable for growing barley, rye and wheat divided by the fraction that was suitable for any crops) and the ancestral suitability of the environment for plough-negative crops (the average fraction of ancestral land that was suitable for growing foxtail millet, pearl millet and sorghum divided by the fraction that was suitable for any crops). In square brackets we report Conley standard errors adjusted for spacial correlation (window = 10 degrees).

***, **, and * indicate significance at the 1%, 5%, and 10% levels.

**Fig 2:**
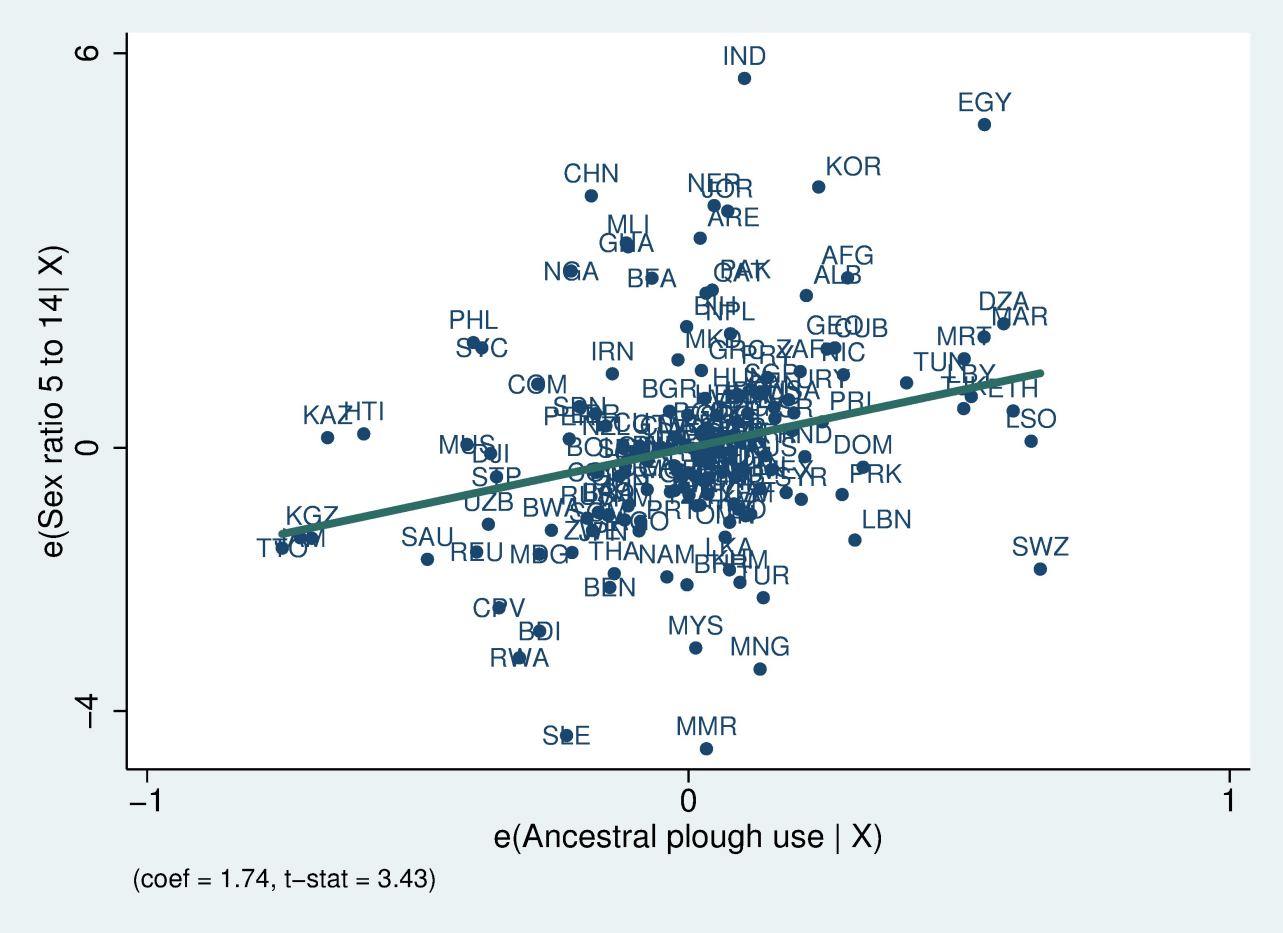
Partial correlation plot: ancestral plough use and the sex ratio of children aged 5 to 14 (boys per 100 girls). The sample includes 153 countries. The sex ratio is calculated as a quinquennial average from 1960–2000. The specification includes continent fixed effects, historical covariates (economic complexity, political hierarchies, the presence of large animals, agricultural suitability and a measure of tropical climate), and contemporaneous covariates (per capita GDP and its square, fertility, and infant mortality). Each country is labeled with its 3-digit iso code.

The estimates show that countries with more ancestral plough use tend to have a higher male-to-female sex ratio. In addition, the estimated effect is systematically larger for children of older age cohorts. Note that the finer age categories are not mutually exclusive. This should be taken into account when comparing the estimates across different dependent variables that measure the sex ratio of different age cohorts. The magnitudes of the estimated coefficients are sizeable. Based upon the estimates from column 1, a one-standard-deviation increase in ancestral plough use (0.44) is associated with an increase of 0.20 standard deviations of the sex ratio measure. For children aged 5–14, the estimated effect of a one-standard-deviation increase in ancestral plough use is 0.33 standard deviations of the sex ratio measure. Columns 5 and 6 of Table 1 report the principal component across the four different sex ratio measures (column 5) and across the sex ratio measures for the two non-overlapping cohorts, 0–4 and 5–14 (column 6). In Fig 2, we report the partial correlation plot for the specification in column 4, where the dependent variable is the sex ratio of children aged 5–14. As shown, the correlation is quite general and not driven by a small number of influential outliers. As part of Supporting Information, we also report the partial correlation plots for other age ranges. The coefficient increases with age (see S6 and S7 Figs).

As an additional measure of the magnitude of the historical plough use, we calculate the change in the *R*-squared when the historical plough use variable is added to the regression equation. For the specification where the dependent variable is the principal component of the sex ratio measures from all cohorts (column 5), the inclusion of the historical plough use variable increases the R-squared by 0.0226. (from 0.5733 to 0.5959). Therefore, a history of plough agriculture accounts for 2.3% of the total variation in the sex ratio’s principal component and 5.3% of the residual variation left unexplained by the control variables [(0.5959–0.5733)/(1–0.5733)]. For comparison, these magnitudes are similar to those from the inclusion of continent fixed effects.

### Instrumental variable regressions

Although our OLS estimates include a wide range of covariates, there remains the concern that omitted factors, correlated with both traditional plough use and the sex ratio today, could be driving the results. To address this, we follow [16] and use instrumental variables to exploit variation in historical plough use that is due to differences in climatic conditions that affected the types of crops that could be grown in a location, and whether they were crops that significantly benefitted from the introduction of the plough or not.

With the assumption that the difference between ancestral suitability for plough-positive and plough-negative crops is only correlated with gender role attitudes today through their impacts on the plough (conditional on covariates), we can use the two measures as instruments in an IV strategy that provides causal estimates of the effect of ancestral plough use on the sex ratio today. We believe that the exclusion restriction is likely satisfied, especially given that in all specifications, we control for the proportion of land historically inhabited by an ethnic group that was tropical or subtropical, as well as its overall agricultural suitability. We also include the same large set of ancestral and contemporaneous controls as is included in the multivariate OLS regressions.

## Results and discussion

The IV estimates are reported in Panel B and C of Table 1, where we report the same set of specifications as for the OLS estimates. Panel C reports the first-stage estimates, where ancestral plough use is the dependent variable and plough-positive crop suitability and plough-negative crop suitability serve as instruments. The estimates show that while plough positive suitability is positively associated with the adoption of the plough, this is not true for plough-negative crop suitability. Thus, the estimates provide confirmation for Pryor’s hypothesis that crop type affected the adoption of the plough. In Tables D and E in S1 Supplementary Material, we examine the stability of these findings over the period of our analysis, 1960–2010. For simplicity, we report the results for the two non-overlapping sex ratios (age 0–4 and 5–14). The importance of the plough appears to be very stable and shows only a very small decline over time. They also provide confirmation about the relevance of our instruments for plough adoption.

The second-stage IV estimates are reported in Panel B of Table 1. They show that ancestral plough use is associated with higher male-to-female sex ratios. This provides evidence that the positive relationship between traditional plough use and the sex ratio, that was estimated using OLS, is not due to omitted variables bias or reverse causality and, thus, is likely causal. As with OLS, the IV estimates are sizeable in magnitude and show that the magnitude of the estimated effect is larger for older age cohorts. For example, based upon the estimates from column 4 and 5, a one-standard-deviation increase in traditional plough use (0.45) is associated with a 0.44 standard-deviation increase in the sex ratio of children aged 0–4 and a 0.58 standard-deviation increase in the sex ratio of children aged 5–14. Columns 5 and 6 of Table 1 report the principal component for the four different sex ratios (column 5) and for the 0–4 and 5–14 sex ratios (column 6). The results are qualitatively similar when we examine these aggregated measures.

Comparing Panels A and B of Table 1, we see that the magnitudes of the IV estimates are consistently larger than the OLS estimates. This is most likely due to a bias towards zero in the OLS estimates, which arises due to the fact that plough societies tend to be more economically advanced both in the past and today. If there is a positive relationship between economic development and equality of gender norms, and if our controls for historical and contemporary income are imperfect, then our OLS estimates will be biased towards zero, while the IV estimates will not suffer from this bias.

There are a number of potential limitations that should be kept in mind in interpreting our results. The primary is that although our procedure, which links historical ethnic groups to contemporary populations using languages, works well for many countries of the world, it works less well for countries where languages have been adopted by other groups within the country. An example is the United States, where all populations speaking English today are assumed to have ancestors that are English, even though we know that many ancestors of English-speakers within the U.S. were from non-English-speaking backgrounds, e.g., Italian, German, etc. To check the importance of this issue for our estimates, we have re-estimated our baseline regressions after omitting all countries from North and South America, as well as Australia, New Zealand and South Africa. The estimates, which are reported in Table F in S1 Supplementary Material, show that our estimates are very similar when we remove these countries from our sample.

A second potential concern relates to the non-independence of ethnicities in the *Ethnographic Atlas*, which has the potential to result in non-independence of observation in our country-level in our sample (i.e., Galton’s problem) [44]. We have addressed the issue of the non-independence of observations by also reporting Conley standard errors, which correct for non-independence of observations in our sample. The correction requires information on the distance between observations. For our baseline estimates, we have used the spatial distance, measured as the average distance between the traditional location of a country’s ancestors, which was calculated using the latitude and longitude of ethnic groups (as reported in the *Ethnographic Atlas*). An alternative strategy is to use genetic distance between countries [45]. Standard errors using this method are very similar to those calculated using geographical distance. These are reported in Table A in S1 Supplementary Material. The Conley correction also requires an assumption about the range of distances for which the correlation between observations is non-zero. Table A in S1 Supplementary Material reports calculated standard errors for different assumptions about this range. Overall, we find that the Conley standard errors, which correct for non-independence of observations, tend to be slightly smaller than conventional standard errors when we use geography and slightly larger when we use genetic distance. Overall, adjusting for non-independence does not alter our statistical inference nor our conclusions.

The third concern is related to the geographical variation in plough use. There is little variation within Europe and within sub-Saharan Africa. Therefore, our estimates could simply be driven by broad differences between continents. All of our estimates include continent fixed effects. To also rule out the possibility that we are not capturing differences between Sub-Saharan Africa and the rest of the world we also disaggregate the African continent indicator into an indicator for sub-Saharan Africa and one for North Africa. The estimates are robust to this additional specification (Table G in S1 Supplementary Material).

The fourth potential concern is that for our instrumental variable strategy, the difference between plough-positive and plough-negative environments may be correlated with geographic features that affect gender attitudes today through channels other than the plough. Although we have controlled for an extensive set of geographical and historical variables, which mitigates this concern, the violation of the exclusion restriction remains a possibility.

The final caveat is that our estimates only reflect the effects of having a tradition of plough agriculture versus not. Ploughs that were adopted varied significantly in size and complexity, and these may have had very different effects on gender roles and, thus, on the sex ratio today. Our analysis is only able to report the average effect of plough adoption across all of the types of ploughs that were adopted.

## Conclusions

Our findings show that the variation in sex ratios that we observe today has deep historical roots. We find that descendants of societies that traditionally practiced plough agriculture have higher average male-to-female sex ratios today. The evidence is consistent with traditional plough use shaping the value placed on boys relative to girls, which continues to persist until today, affecting observed sex ratios.

## Supporting information

S1 Fig Distribution of sex ratios for different age groups. The figure shows the distribution of sex ratio by age groups.
(TIF)

S2 Fig Population density and language groups within Ethiopia. The figure shows a map of the land inhabited by different language groups, i.e. groups speaking different languages or dialects. Each polygon represents the approximate borders of a group (from *Ethnologue*). A darker background shade indicates a more densely populated area. These data are from *Landscan*.
(TIF)

S3 Fig Language groups and traditional plough use within Ethiopia. The figure show which of the language groups (from *Ethnologue*) have ancestors that used the plough (this information is taken from the *Ethnographic Atlas*).
(TIF)

S4 Fig Ancestral plough use across countries. The figure shows the fraction of each country’s population with ancestors that used the plough in pre-industrial agriculture.
(TIF)

S5 Fig Partial correlation plot: ancestral plough use and the sex ratio at birth (boys per 100 girls). The sample includes 153 countries. The sex ratio is a quinquennial average from 1960–2000. The specification includes continent fixed effects, historical covariates (economic complexity, political hierarchies, the presence of large animals, agricultural suitability and a measure of tropical climate), and contemporaneous covariates (per capita GDP and its square, fertility, and infant mortality). Each country is labelled with its 3-digit iso code.
(TIF)

S6 Fig The partial correlation between ancestral plough use and sex ratio between ages 0 and 1, after the inclusion of continent fixed effects, and historical and contemporaneous controls. The graph shows the correlation between historical plough use and sex ratio between ages 0 and 1 (boys per 100 girls) for a sample of 153 countries during the period 1960–2000, after controlling for average differences in the sex ratio between continents, historical country differences (including economic complexity, political hierarchies, the presence of large animals, agricultural suitability and a measure of tropical climate) and contemporaneous measures of per capita GDP, fertility and infant mortality. Each country is labelled with its 3-digit iso code.
(TIF)

S7 Fig The partial correlation between ancestral plough use and sex ratio between ages 0 and 4, after the inclusion of continent fixed effects, and historical and contemporaneous controls. The graph shows the correlation between historical plough use and the sex ratio between ages 0 and 4 (boys per 100 girls) for a sample of 153 countries during the period 1960–2000, after controlling for average differences in the sex ratio between continents, historical country differences (including economic complexity, political hierarchies, the presence of large animals, agricultural suitability and a measure of tropical climate) and contemporaneous measures of per capita GDP, fertility and infant mortality. Each country is labelled with its 3-digit iso code.
(TIF)

S1 Supplementary Material Supplementary Material for traditional agricultural practices and the sex ratio today. The Supplementary Material provides details on the data sources and the robustness of the analysis for the results derived in the paper.
(DOCX)

S1 File Replication package. This zip file contains the underlying dataset and the STATA do-file used to replicate the results of the manuscript.
(ZIP)
