# Supplementary figures and images for: Traditional agricultural practices and the sex ratio today
Source: PLoS One. 2018 Jan 16;13(1):e0190510. doi: 10.1371/journal.pone.0190510 (PMC5770021; doi:10.1371/journal.pone.0190510)

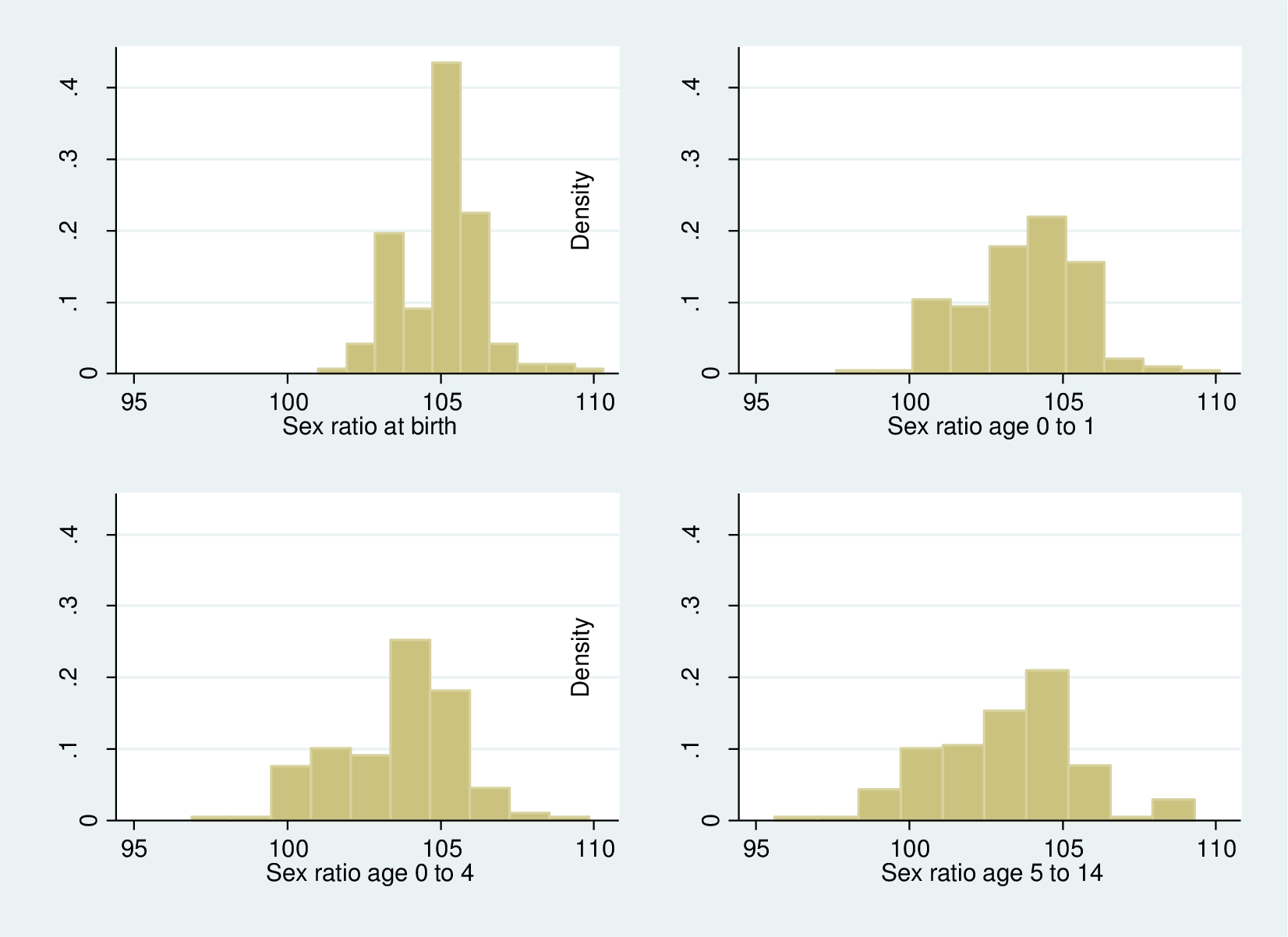

Supplement: S1 Fig — The figure shows the distribution of sex ratio by age groups. (TIF) [file pone.0190510.s001.tif]

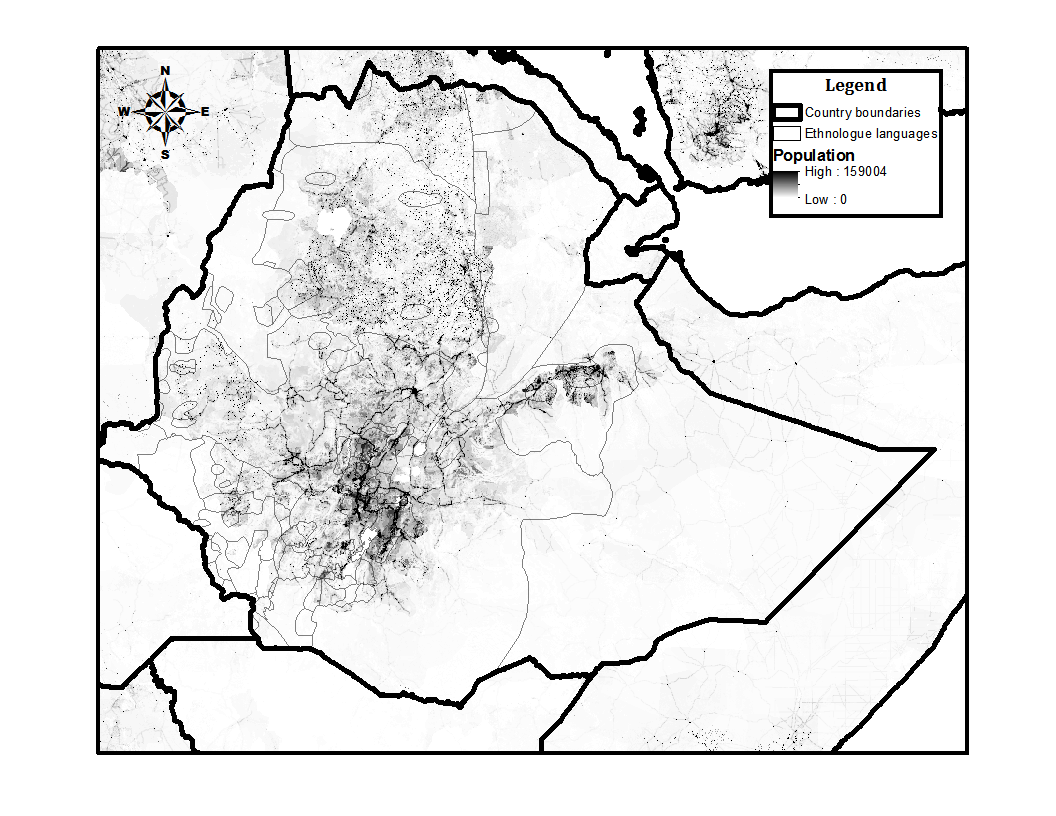

Supplement: S2 Fig — The figure shows a map of the land inhabited by different language groups, i.e. groups speaking different languages or dialects. Each polygon represents the approximate borders of a group (from Ethnologue). A darker background shade indicates a more densely populated area. These data are from Landscan. (TIF) [file pone.0190510.s002.tif]

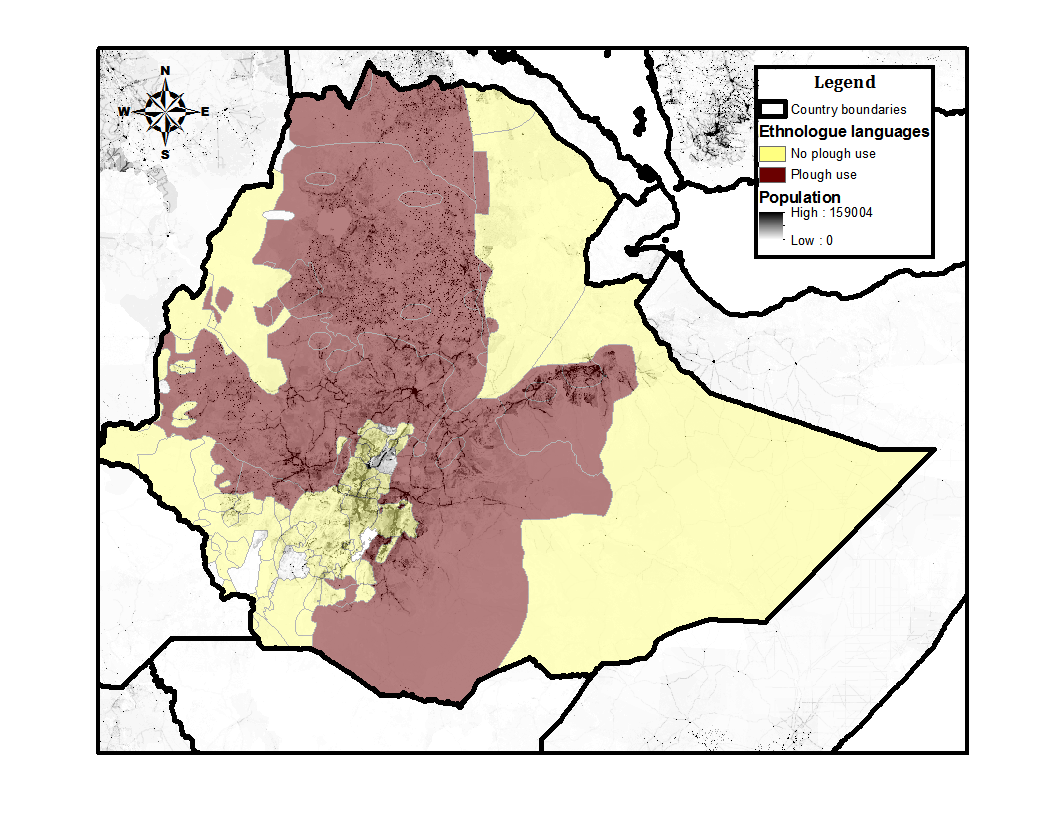

Supplement: S3 Fig — The figure show which of the language groups (from Ethnologue) have ancestors that used the plough (this information is taken from the Ethnographic Atlas). (TIF) [file pone.0190510.s003.tif]

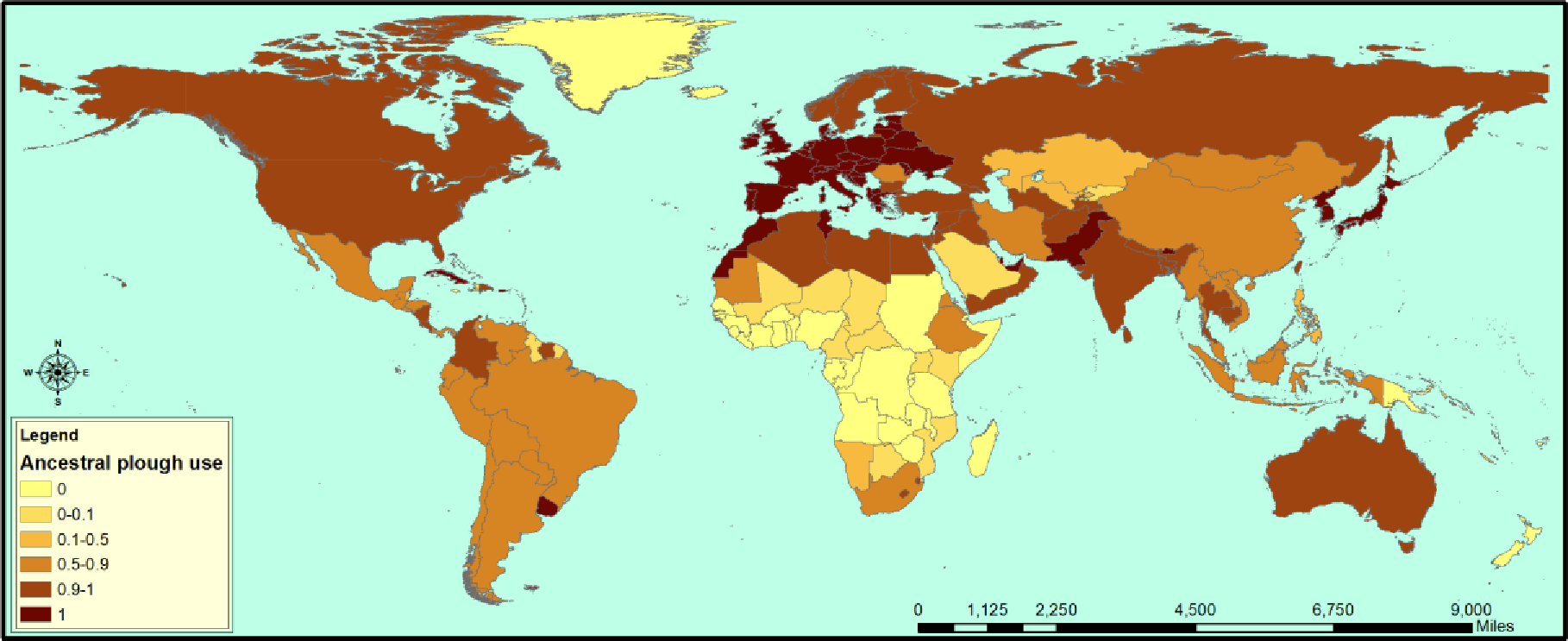

Supplement: S4 Fig — The figure shows the fraction of each country’s population with ancestors that used the plough in pre-industrial agriculture. (TIF) [file pone.0190510.s004.tif]

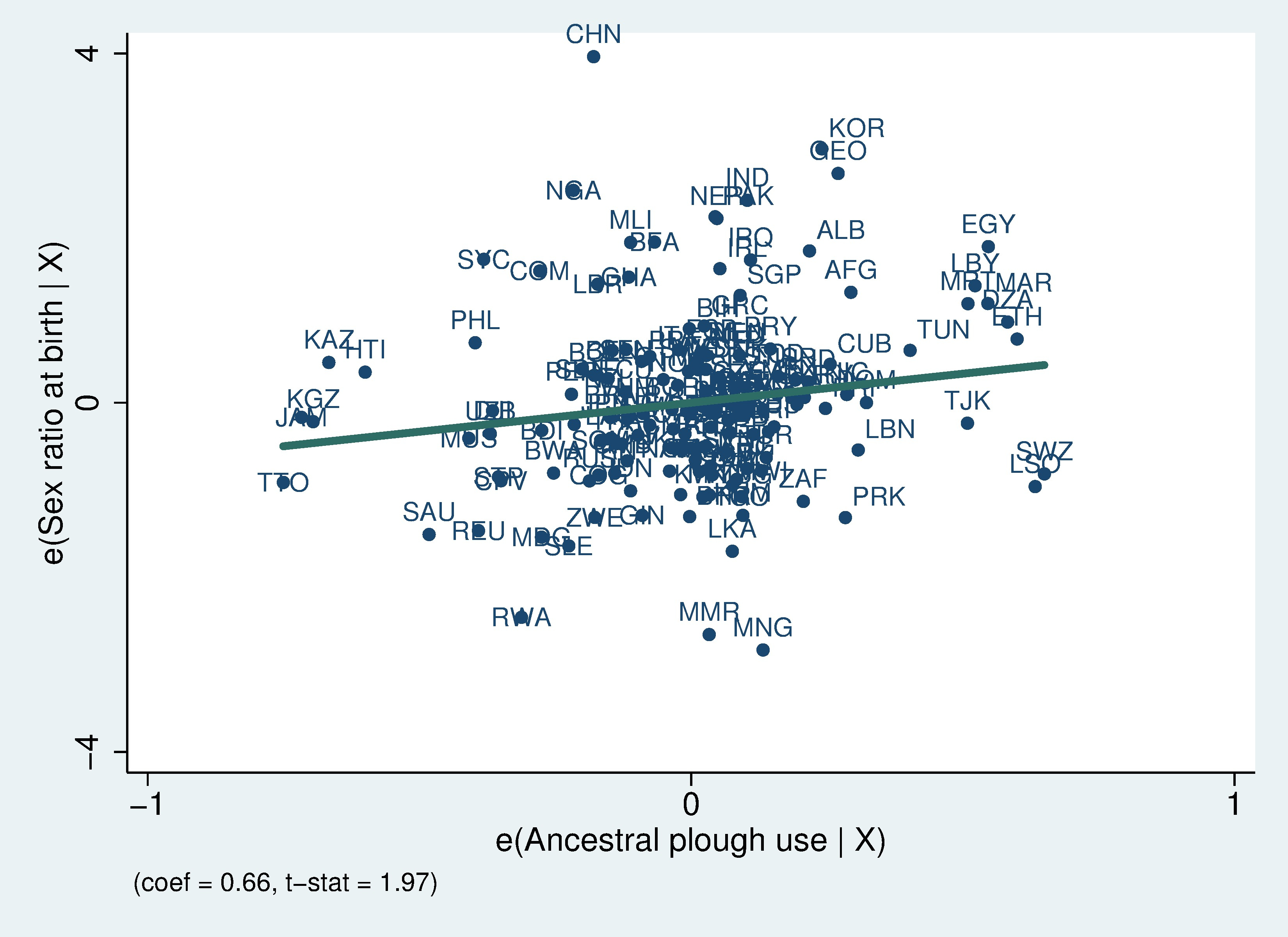

Supplement: S5 Fig — The sample includes 153 countries. The sex ratio is a quinquennial average from 1960–2000. The specification includes continent fixed effects, historical covariates (economic complexity, political hierarchies, the presence of large animals, agricultural suitability and a measure of tropical climate), and contemporaneous covariates (per capita GDP and its square, fertility, and infant mortality). Each country is labelled with its 3-digit iso code. (TIF) [file pone.0190510.s005.tif]

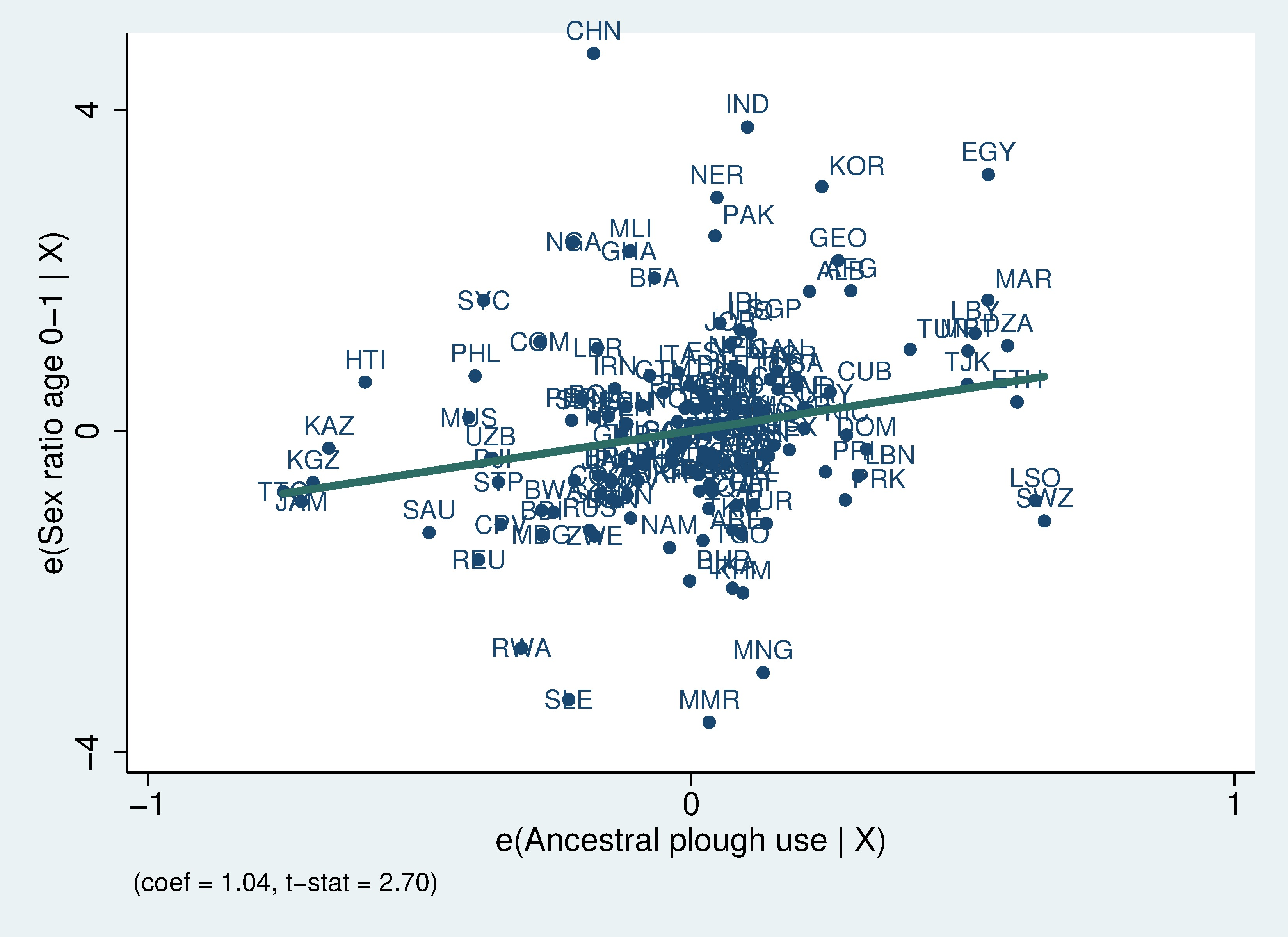

Supplement: S6 Fig — The graph shows the correlation between historical plough use and sex ratio between ages 0 and 1 (boys per 100 girls) for a sample of 153 countries during the period 1960–2000, after controlling for average differences in the sex ratio between continents, historical country differences (including economic complexity, political hierarchies, the presence of large animals, agricultural suitability and a measure of tropical climate) and contemporaneous measures of per capita GDP, fertility and infant mortality. Each country is labelled with its 3-digit iso code. (TIF) [file pone.0190510.s006.tif]

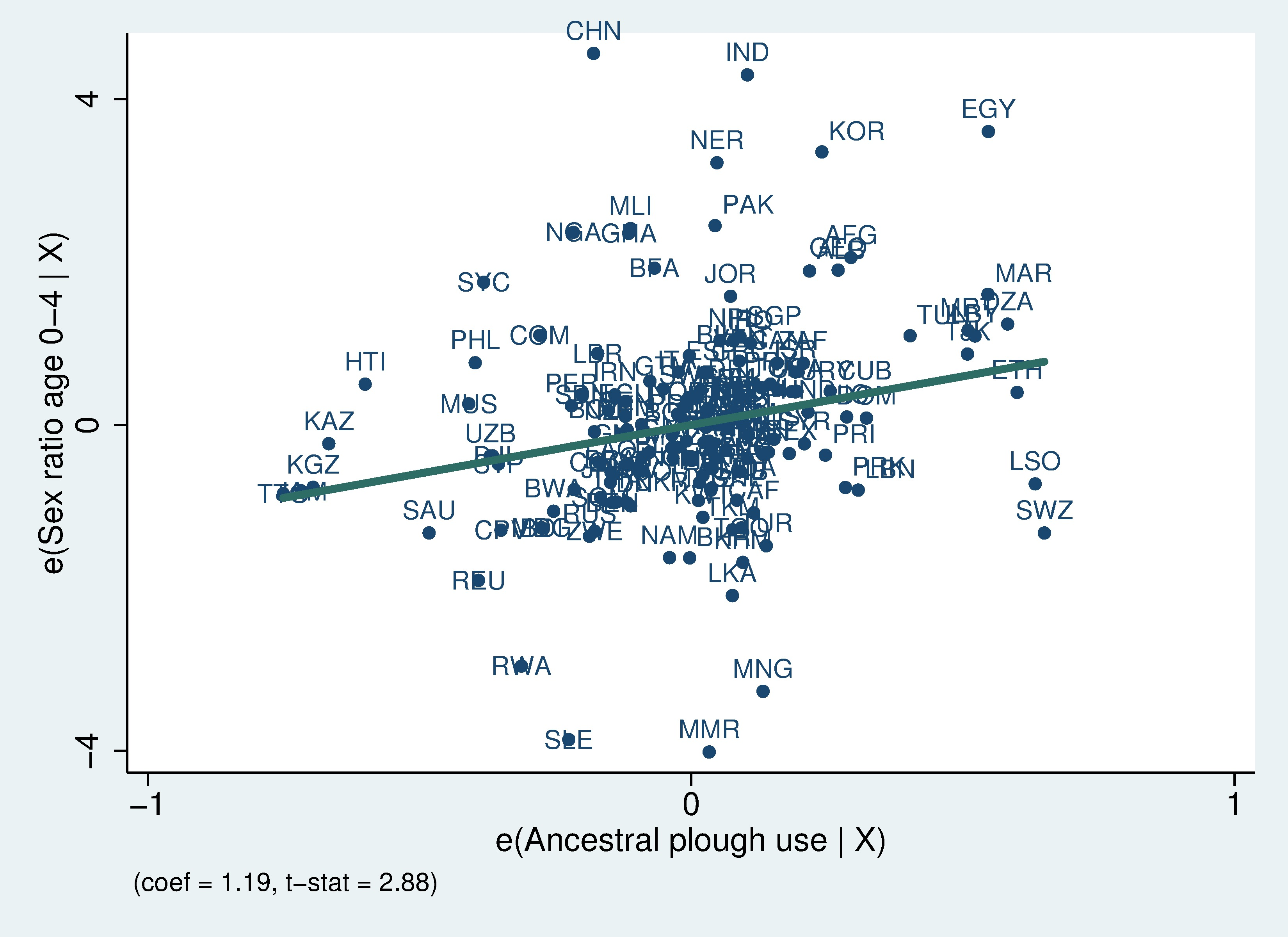

Supplement: S7 Fig — The graph shows the correlation between historical plough use and the sex ratio between ages 0 and 4 (boys per 100 girls) for a sample of 153 countries during the period 1960–2000, after controlling for average differences in the sex ratio between continents, historical country differences (including economic complexity, political hierarchies, the presence of large animals, agricultural suitability and a measure of tropical climate) and contemporaneous measures of per capita GDP, fertility and infant mortality. Each country is labelled with its 3-digit iso code. (TIF) [file pone.0190510.s007.tif]
