# Supplementary material for: Traditional agricultural practices and the sex ratio today
Source: PLoS One. 2018 Jan 16;13(1):e0190510. doi: 10.1371/journal.pone.0190510 (PMC5770021; doi:10.1371/journal.pone.0190510)
Supplement: S1 Supplementary Material — The Supplementary Material provides details on the data sources and the robustness of the analysis for the results derived in the paper. (DOCX) [file pone.0190510.s008.docx]

Supplementary Material for

Traditional agricultural practices and the sex ratio today

Alberto Alesina^1,2^, Paola Giuliano^3,2,^*, Nathan Nunn^1,2^

^1^ Harvard University.

^2^ NBER.

^3^ UCLA.

*Correspondence to: paola.giuliano@anderson.ucla.edu

The Supplementary Material provides details on the data sources and the robustness of the analysis for the results derived in the paper.

**Additional details on the *Ethnographic Atlas***

The *Ethnographic Atlas* was constructed by George Peter Murdock. It contains ethnographic information for 1,265 ethnic groups worldwide. The period in which the information was collected varies by ethnicity, with the earliest observation dates coming from ethnicities in the Old World (where early written evidence is available) and the most recent information dating around the 20^th^ century, for those parts of the world without a written history and directly observed by anthropologists. All societies are observed prior to industrialization. In total, 23 ethnicities are observed during the 17^th^ century or earlier, 16 during the 18^th^ century, 310 during the 19^th^ century, 876 between 1900 and 1950, and 31 after 1950. For nine ethnicities, an exact year is not provided.

The variable *v*39 classifies each ethnic group as being in one of the following three categories: (1) the plough was absent, (2) the plough existed at the time the group was observed, but it was not aboriginal, and (3) the plough was aboriginal, having existed prior to contact. Using this information, we construct an indicator variable that equals one if the plough was ever adopted during the pre-industrial period (whether aboriginal or not) and zero otherwise. Of the 1,156 ethnicities for which information exists, for 997 the plough was absent, for 141 the plough was adopted (and aboriginal), and for 18 it was adopted, but after European contact. Of these, almost all are Native American groups who today are either extinct or are very small and comprise a negligible proportion of the population of countries today. The exceptions are the Swazi, Sotho, and Xhosa, all from the Southern part of Africa and the Aymara who live in Bolivia today. After our data construction procedure, there are only four countries with non-trivial proportions of the population whose ancestors adopted the plough after European contact. These are Swaziland (99.9%), Lesotho (97.3%), Bolivia (30.55%) and South Africa (23%). We report in S2 and S3 Tables the robustness of the results to the exclusion of the four countries where a large proportion of the population adopted the plough after European contact, and the twenty-one for which there is any proportion of the population for which the plough was not aboriginal. The results are more precisely estimated when compared to the full sample.

**Table A. Sex ratio ancestral plough use, robustness to Conley standard errors.**

**Table B. Sex ratio and ancestral plough use, robustness to the exclusion of countries with a large fraction of the population with non-aboriginal plough use (Swaziland, Lesotho, Bolivia and South Africa).**

**Table C. Sex ratio and ancestral plough use, robustness to the exclusion of 21 countries with any fraction of the population with of non-aboriginal plough use.**

**Table D. Sex ratio between ages 0 and 4 and ancestral plough use.**

**Table E. Sex ratio between ages 5 and 14 and ancestral plough use.**

**Table F. Sex ratio and ancestral plough use, excluding Australia, New Zealand, South Africa, North and South America.**

**Table G. Sex ratio and ancestral plough use, including a dummy for Sub-Saharan Africa.**

**Table H. Sex ratio and ancestral plough use, list of countries included in Table 1.**
